# Clinical practice recommendations for allergen-specific immunotherapy in children: the Italian consensus report

**DOI:** 10.1186/s13052-016-0315-y

**Published:** 2017-01-23

**Authors:** Giovanni Battista Pajno, Roberto Bernardini, Diego Peroni, Stefania Arasi, Alberto Martelli, Massimo Landi, Giovanni Passalacqua, Antonella Muraro, Stefania La Grutta, Alessandro Fiocchi, Luciana Indinnimeo, Carlo Caffarelli, Elisabetta Calamelli, Pasquale Comberiati, Marzia Duse, Allergen-specific Immunotherapy panel of the Italian Society of Pediatric Allergy and Immunology (SIAIP), Cezmi Akdis, Mübeccel Akdis, Sergio Arrigoni, Salvatore Barberi, Giuseppe Baviera, Attilio Boner, Mauro Calvani, Luigi Calzone, Lucia Caminiti, Angelo Capristo, Carlo Capristo, Fernanda Chiera, Claudio Cravidi, Giuseppe Crisafulli, Giovanna De Castro, Monica De Simone, Iride dello Iacono, Arianna Dondi, Elena Galli, Francesco Guglielmo, Nunzia Maiello, Gianluigi Marseglia, Paolo Maria Matricardi, Paolo Meglio, Domenico Minasi, Michele Miraglia del Giudice, Girolamo Panasci, Francesco Paravati, Umberto Pelosi, Giuseppe Pingitore, Giampaolo Ricci, Guglielmo Scala, Luigi Terracciano, Mariangela Tosca, Salvatore Tripodi, Maria Carmen Verga, Ulrich Wahn

**Affiliations:** 1 Department of Pediatrics, Allergy Unit, University of Messina, Via Consolare Valeria-Gazzi, Messina, 98124 Italy; 2 Pediatric Unit, Hospital “Nuovo Ospedale S. Giuseppe”, Empoli, Italy; 3 Pediatric Unit, University of Pisa, Pisa, Italy; 4 Molecular Allergology and Immunomodulation- Department of Pediatric Pneumology and Immunology, Charité Medical University Berlin, Berlin, Germany; 5 Pediatric Unit, Hospital “G. Salvini”, Garbagnate Milanese, Italy; 6 National Pediatric Healthcare System, Turin, Italy; 7 National Research Council of Italy, Institute of Biomedicine and Molecular Immunology, Palermo, Italy; 8 Allergy and Respiratory Diseases, IRCCS San Martino-IST-University of Genoa, Genoa, Italy; 9 Food Allergy Referral Centre Veneto Region, Department of Women and Child Health, Padua General University Hospital, Padua, Italy; 10 Bambino Gesù Pediatric Hospital, Rome, Italy; 11 Department of Pediatrics, University “La Sapienza”, Rome, Italy; 12 Pediatric Unit, Department of Clinical and Experimental Medicine, University of Parma, Parma, Italy; 13 Pediatric Unit, University of Bologna, Bologna, Italy; 14 Pediatric Unit, University of Verona, Verona, Italy

**Keywords:** Allergy, Asthma, Atopic dermatitis, Children, Food allergy, Allergen-specific immunotherapy, Sub-lingual immunotherapy, Subcutaneous immunotherapy

## Abstract

Allergen-specific immunotherapy (AIT) is currently recognized as a clinically effective treatment for allergic diseases, with a unique disease-modifying effect. AIT was introduced in clinical practice one century ago, and performed in the early years with allergenic extracts of poor quality and definition. After the mechanism of allergic reaction were recognized, the practice of AIT was refined, leading to remarkable improvement in the efficacy and safety profile of the treatment. Currently AIT is accepted and routinely prescribed worldwide for respiratory allergies and hymenoptera venom allergy. Both the subcutaneous (SCIT) and sublingual (SLIT) routes of administration are used in the pediatric population.

AIT is recommended in allergic rhinitis/conjunctivitis with/without allergic asthma, with an evidence of specific IgE-sensitization towards clinically relevant inhalant allergens. Long-term studies provided evidence that AIT can also prevent the onset of asthma and of new sensitizations. The favorable response to AIT is strictly linked to adherence to treatment, that lasts 3–5 years. Therefore, several factors should be carefully evaluated before starting this intervention, including the severity of symptoms, pharmacotherapy requirements and children and caregivers’ preference and compliance.

In recent years, there have been increasing interest in the role of AIT for the treatment of IgE-associated food allergy and extrinsic atopic dermatitis. A growing body of evidence shows that oral immunotherapy represents a promising treatment option for IgE-associated food allergy. On the contrary, there are still controversies on the effectiveness of AIT for patients with atopic dermatitis.

This consensus document was promoted by the Italian Society of Pediatric Allergy and Immunology (SIAIP) to provide evidence-based recommendations on AIT in order to implement and optimize current prescription practices of this treatment for allergic children.

## Background

**Fig. 1:**
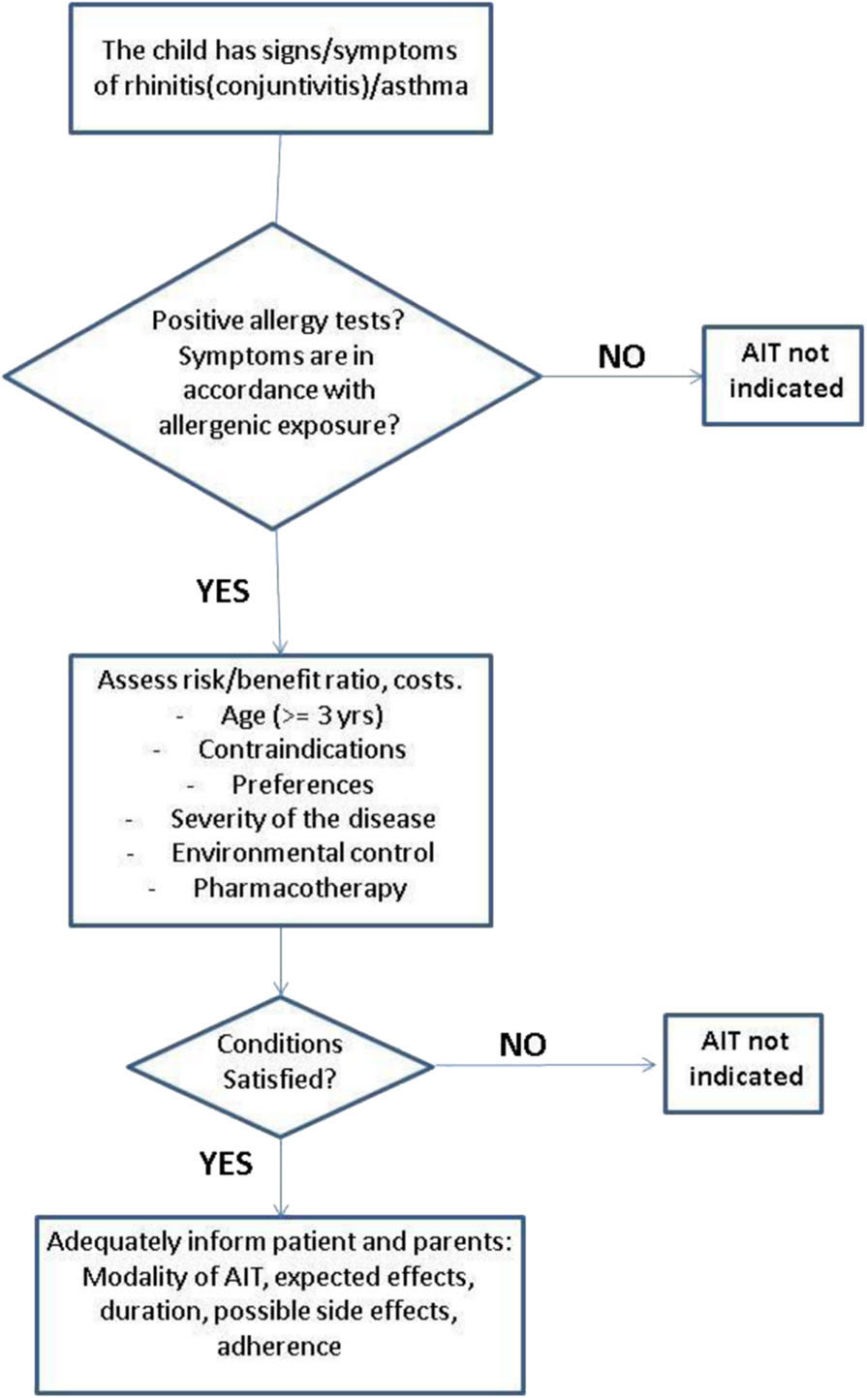
Proposed algorithm

This consensus document was promoted by the Italian Society of Pediatric Allergy and Immunology (SIAIP) to implement the effective and safe use of AIT in children with allergic diseases and to optimize clinical practice recommendations for this treatment. A large number of experts in the field of pediatric allergy and immunology (academicians and territorial pediatricians) equally contributed to this manuscript, that summarizes the SIAIP consensus conference on AIT held in October 2013 in Lipari, Italy, and updates the previous document [1, 2]. A comprehensive search of the medical literature was carried out. References were identified by searches of PubMed and online Cochrane databases. The search strategies used the major keywords “allergy” and “immunotherapy” and all of the following, separately and in combination: asthma/children/epicutaneuous immunotherapy/food allergy/management/oral immunotherapy/rhinitis/rhino-conjunctivitis/treatment/subcutaneous immunotherapy/sublingual immunotherapy. We reviewed, analyzed and considered only those studies published in English language and involving pediatric populations (age 0–18 years), published up to May 2016. The published clinical trials were assessed by category of evidence and used to determine the strength of recommendations (Table 1), also according to other international documents [3–6]. An algorithm summarizes the key issues for the effective use of AIT in allergic children (Fig. 1). The specific recommendations indicate how to select eligible children for AIT, provided that an IgE sensitization towards one or more relevant allergens is documented, associated with consistent symptoms of allergic rhinitis/conjunctivitis and/or allergic asthma.

**Table 1:** Classification of evidence for allergen-specific immunotherapy used in the manuscript

Level of evidence	
Ia Evidence from meta-analysis of randomized controlled studies Ib Evidence from at least one randomized controlled study IIa Evidence from well-designed controlled trial without randomization IIb Evidence from at least one other type of experimental study III Evidence from non-experimental descriptive studies, including retrospective, case-control studies, cohort studies with controls IV Evidence form opinions of respected authorities, based on clinical experience, or reports of expert committees	
Grades of recommendations	
A: Directly based on Level I evidence. A certain therapeutic intervention is strongly recommended. B: Directly based on Level II evidence or extrapolated recommendations from Level I evidence. A certain therapeutic intervention should be carefully considered. C: Directly based on Level III or IV evidence or extrapolated recommendations from Level I or II evidence. There is significant uncertainties about a certain therapeutic intervention.

AIT can induce long-term effects after discontinuation of the treatment and can modulate the natural course of allergic disease by preventing the onset of new sensitizations and the progression of respiratory allergies [7]. In food allergy, although the available studies described an effective clinical desensitization in the majority of patients, this approach remains so far an experimental strategy [8]. The same holds true for atopic dermatitis (AD) associated with IgE-sensitization to inhalant allergens [9]. This document also contains specific recommendations for the prevention and therapeutic management of adverse effects caused by AIT.

SCIT and SLIT are currently accepted as effective treatments respiratory allergic diseases in children, and some SLIT products were approved as drugs by EMA and FDA [10, 11]. Concerning hymenoptera venom allergies, this document does not analyze this issue, please refers to the most recent and specific guidelines for adults [12] and to the document developed by SIAIP in 2010 [2].

This consensus report provides recommendations and suggestions to specialists for the use of AIT in pediatric allergy, as derived by current literature. The document does not replace, anyway, the clinical judgment that has to be applied to each individual patient.

## Choice of relevant allergens for allergen-specific immunotherapy products

The European Medicines Agency (EMA) defines relevant allergens as “allergens causing clinically relevant effects in a significant proportion of allergic patients” [13, 14]. The presence of relevant allergens in the commercial preparations should be documented by appropriate methods of quantification as the antibody techniques or the mass spectrometry. The antibody techniques for identifying the concentration of relevant allergens are the following: the radioimmunoassay (RIA), the enzyme-linked immunosorbent assay (ELISA), the radial immunodiffusion (RID), or the rocket Immunoelectrophoresis [15–17]. The major allergens are reported in Table 2.

**Table 2:** The major genuine sensitizers from the relevant allergenic sources

Allergen source	Species	Specific allergen molecules
House dust mite	Dermatophagoides pteronyssinus	Der p 1 Der p 2
	Dermatophagoides Farinae	Der p 23 Der f 1
	Blattella Germanica (cockroach)	Der f 2 Bla g 1
Pet dander	Felis domesticus (Cat)	Fel d 1, Fel d 2
	Canis familiae (Dog)	Can f 1, Can f 3
Grass Pollen	Phleum pratense (Timothy grass)	Phl p 1, Phl p 5, Phl p 6
	Cynodon dactylon (Bermuda grass)	Cyn d 1
	Lolium perenne (Ryegrass)	Lol p 1, Lol p 5
	Poa pratensis (Meadow)	Poa p 1, Poa p 5
	Dactylis glomerata (Cocksfoot)	Dac g 1, Dac g 5
	Holcus lanata (Velvet grass)	Hol l 1, Hol l 5
Tree Pollen	Betula verrucosa (Birch)	Bet v 1
	Olea europoea (Olive)	Ole e 1
	Alnus glutinosa (Alder)	Aln g 1
	Cryptomeria Japonica (Japanese cedar)	Cry j 1
	Cupressus arizonica (Cypress)	Cup a 1
Weed pollen	Ambrosia artemisifolia (Ragweed)	Amb a 1
	Artemisia vulgaris (Mugwort)	Art v 1
	Parietaria judaica (Wall pellitory)	Par j 1 Par j 2
Molds	Alternaria alternata	Alt a1
	Aspergillus Fumigatus	Asp f 1
	Cladosporium herbarum	Cla h 1

Grass pollen allergens have a high cross-reactivity. Phl p 1 (Phleum pratense–Timothy grass) has similar reactivity with Lol p 1 (Lolium perenne–Ryegrass), Poa p 1 (Poa pratensis–Meadow grass), Dac g 1 (Dactylis glomerata–Cocksfoot) and Hol l 1 (Holcus lanatus–Velvet grass). The allergen content of each preparation used for AIT should contain from 5 to 20 mcg of the major or prevalent allergen for SCIT and 15–187 mcg for SLIT [18]. Chemically modified allergens are named “allergoids”: these products maintain the desired immunogenicity, with a reduced allergenicity. The chemical modification can be carried out both with molecules binding allergens (formaldehyde, glutaraldehyde, alginate) and with molecules that substitute a functional group with another (potassium cyanate). This reaction called carbamylation of allergenic extracts [19] confers to the product a reduced IgE affinity and a partial resistance to the proteolytic enzymes in the digestive system. Modifications also include physical changes, thus chemically modified allergens can be adsorbed onto tyrosine, alum salts or alginate. Because of their relevance, allergens should be accurately quantified in preparations used for AIT. Currently, each manufacturing company has standard in-house reference extracts with known biological activity, with a relevant variability in allergen content [14, 20, 21]. This condition remains one of the main limits of AIT. Nowadays, preparations with a high concentration, well tolerated and safe, have been included in the official pharmacopoeia, for which the concentration of major allergens is provided in mcg/ml. In this way, patients would be treated with adequately standardized extracts.

The use of molecular diagnosis techniques [22] may allow physicians to better identify whether children with allergic respiratory symptoms are sensitized to major allergens or to crossreactive molecules [23–25]. This is of particular interest in patients who are sensitized to several pollens, to prescribe AIT only for major allergens [24, 25]. with the aim to increase the effectiveness of AIT and to better select patients who need a treatment. Furthermore, the molecular diagnostics could be a suitable instrument to implement the early therapeutic intervention with AIT in children with respiratory allergy.

## Allergen specific immunotherapy for respiratory allergic diseases

### Subcutaneous immunotherapy (SCIT)

Over the last 50 years, numerous controlled studies have shown the efficacy of SCIT in the treatment of allergic asthma and rhinitis [26, 27]. These data have been evaluated in several meta-analyses and reviews. A Cochrane analysis [28] included 51 studies for a total of 2871 participants (1645 active, 1226 placebo), who underwent AIT for seasonal allergic rhinitis for a duration from 3 months to 5 years. The meta-analysis showed a significant clinical improvement in the AIT groups both in the symptom score (data from 15 trials) and in the medication score (data from 13 studies). It was also shown an improvement of immunological parameters and in the quality of life. The meta-analysis excluded studies on pediatric age, but in six studies were included also patients aged under 18 years, even if no specific outcomes were evaluated in the pediatric population.

The efficacy of SCIT for the treatment of asthma was evaluated in a meta-analysis including 101 studies (3792 patients) carried-out both in adults and in children but no sub-analysis of the specific outcomes in the pediatric groups was performed [29]. In particular, 42 studies of AIT involved patients with mite allergy, 27 pollen allergy (mostly grasses), 10 animal dander allergy, 2 Cladosporium allergy, two latex allergy and six patients with multiple aeroallergens allergy. A significant reduction of symptoms was found in patients treated with mite and pollen AIT, while no significant improvement was recorded for animal dander or allergenic mixtures. Despite the heterogeneity of the included studies, the overall reduction of symptoms (for all allergens) was significant (SMD −0.59, 95% CI −0.83 to −0.35). The medication scores and the bronchial hyper-reactivity were significantly reduced, too. Out of the enrolled subjects, most of them with mild to moderate asthma and normal baseline spirometric parameters, no significant effect on lung function was detected.

A systematic review, including RCTs on AIT for the treatment of pediatric asthma and rhinitis [30], evaluated 13 studies on SCIT, for a total number of 920 children suffering from asthma (7 studies), asthma with rhinitis/rhino-conjunctivitis (5 studies) and rhino-conjunctivitis (1 study). The allergens evaluated were house dust mites in 8 of 13 studies, while in the remaining pollens and moulds. Six RCTs including 550 patients evaluated the effectiveness of SCIT on asthma symptoms, compared to placebo or to pharmacotherapy. Studies with single-allergen SCIT have shown efficacy in reducing symptoms (moderate evidence), while multiple allergens SCIT (1 study) did not improve symptoms in subjects with moderate to severe asthma, except in younger children aged between 5 and 8 years. The effect on the use of medication was evaluated in four studies for asthma and in two for asthma with rhino-conjunctivitis. In all studies it was detected a reduction in the use of medications. Three studies, including 285 subjects suffering from allergic rhinitis, showed an overall improvement of symptoms with a moderate to strong level of evidence. Another review [31] analyzed 31 studies involving patients aged 3–18 years receiving SCIT for mites, grasses, birch or Alternaria. The quality of the studies was assessed using the Jadad score and the GRADE system [32], and the review process was rigorously conducted, putting together studies with different design: double-blind placebo-controlled (DBPC), randomized clinical trials (RCTs) and observational studies. Four studies on grass pollen AIT provided high quality evidence: SCIT induced a reduction in symptom score, a decrease in skin reactivity and an increase in the threshold of specific nasal and/or bronchial provocation test. Long-term efficacy up to 7 years after the end of treatment was also reported [33]. Studies on Alternaria immunotherapy (3 studies) showed similar results. Concerning mite (22 studies) a reduction in symptoms scores, medication use, skin reactivity and emergency room visits, with an increased quality of life were observed. The effects on the reduction of bronchial hyper-responsiveness and on the improvement in lung function were less significant.

### Sublingual immunotherapy

The available meta-analyses of the efficacy of SLIT in children consistently showed a significant efficacy of the treatment for patients with mild to moderate asthma. Olaiguibel et al. [34] observed that the main effect was the reduction in symptoms, less relevant than for medication usage. The allergen used was mainly mite. A further meta-analysis [35] including nine studies (out of 73 examined) for a total of 441 children aged 3–18 years with asthma (with or without allergic rhinitis) found a significant reduction of both parameters examined: symptoms and medication use. The authors emphasize the considerable heterogeneity related to the methods of evaluation of clinical asthma scores. Another meta-analysis of ten studies including 484 children [36] reported similar results.

The most recent studies of SLIT with birch pollen and with grass pollen [37–39] were performed on a large number of patients adequately randomized, using high-dose preparations. These studies further confirmed the efficacy of SLIT for the treatment of seasonal allergic rhinitis and asthma. Overall, the reviews of the literature on pediatric populations consistently support the efficacy and safety of SLIT compared to placebo [40–42], and only one review reported negative results. However, the latter didn’t include all the eligible studies nor considered all relevant outcomes [43].

#### Recommendation 1

AIT should be considered in pediatric patients with rhino-conjunctivitis and/or asthma with an ascertained IgE-associated respiratory allergy, caused by clinically relevant allergens. The decision to start the treatment depends on several factors: the severity of the allergic disease, the acceptance and adherence to the treatment, the response to environmental prevention measures. AIT should be always used in association with appropriate pharmacotherapy (IaB).

## Selection of patients

AIT should be considered as therapeutic option in children suffering from allergic rhinitis/conjunctivitis with/without allergic asthma. Indeed, most allergic subjects can potentially benefit from AIT, if carefully selected with the appropriate allergy diagnostic tests. Since AIT is allergen-specific, its efficacy and effectiveness depends on a proper identification of the triggering allergen(s). That means a proper recording of the clinical history and ascertainment of environmental exposure, confirmed by diagnostic tests. The prescription of AIT depends also on the severity and duration of symptoms, that should be carefully evaluated individually (Table 3). Essential parameters to assess the severity of the allergic disease (in particular of asthma) are the need of additional specialist visits and of the accesses to the emergency department; the response to pharmacotherapy and to allergen avoidance measures; as well as the recurrence of symptoms impairing school or sport activities or altering sleep quality.

**Table 3:** Indications for allergen-specific immunotherapy (AIT) for pediatric allergic rhinitis, conjunctivitis with/without asthma

AIT should be considered for patients with evidence of specific IgE sensitization towards one or few clinically relevant allergen(s).	
The decision to start AIT depends on various factors including:	
• Children’s (and caregivers) preference and acceptability	
• Adherence to treatment	
• Severity of symptoms and pharmacotherapy requirements	
• Efficacy of avoidance measures (e.g. house dust mites, pollens)	
• Asthma and co-existent rhinitis	
Potential indications:	
• Possible prevention of new sensitizations in mono-sensitized patients	
• IgE-associated food allergy	
• Extrinsic atopic dermatitis	

Before prescribing AIT, asthma symptoms should be well-controlled. In children who can cooperate, the parameters of lung function have to be measured in order to assess the level of asthma control. The prescription of AIT is indicated when FEV1 is greater than 70% compared to the predicted value, and uncontrolled asthma remains the major absolute contraindication [44]. Although there is no current general agreement on the administration of AIT in younger children, some clinical studies have shown the efficacy and safety of both routes of administration (SCIT and SLIT) also in preschool children [45–47]. It should be considered that repeated injections of SCIT can be traumatic in small children, and problems of communication and misunderstanding especially in children aged < 4 years might occur [48]. Therefore, in this age group a careful risk/benefit evaluation should be assessed individually for each patient.

In addition, AIT should be considered in those patients who experienced severe or frequent medications’ side effects or wish to avoid prolonged pharmacotherapy. The pharmaco-economic analysis of studies on cost-effectiveness shows that AIT does not seem more expensive compared to the long-term pharmacotherapy [49, 50].

### Recommendation 2

AIT should be considered as therapeutic option in children suffering from allergic rhinitis/conjunctivitis with/without allergic asthma also in preschool age (IbB).

## Duration and adherence

The recommended duration of the treatment is at least 3 years [3–5]. A single study compared the efficacy of a 3-year versus 5-year course of AIT, showing an overall similar efficacy of the two regimens [51]. Also, in a 15-year study in adults with dust mite allergy, it was shown that a 3-year duration of SLIT conferred a long-lasting effect for five further years after discontinuation, and a 4-year course did slightly better [52].

According to the good clinical practices, it is mandatory to:

- assess the effectiveness of AIT; after the patient has reached the maintenance dose, the clinical improvement should be assessed within a short period.
- check the onset of any side effects;
- assess the adherence to the treatment;
- evaluate possible modifications to the current dosing schedule.

The severity of the disease, the effectiveness and the advantages of the treatment for the allergic children represent relevant criteria to decide if carrying on or discontinuing AIT. The causes underlying a poor or absent improvement of symptoms usually may be: 1) incorrect identification and treatment of the clinically relevant allergens; 2) inadequate dose of allergen administered to the patient; 3) too short duration of the therapy; and 4) poor adherence to treatment. As opposite, once assessed the effect of immunotherapy, the treatment should be continued for not less than 3 years. The duration of the treatment may be prolonged (5 years or more), depending on the clinical response of subjects. Many patients experience a prolonged remission of symptoms after discontinuation of AIT [52–55] whereas others may have a relapse of clinical manifestations. Currently, there are no specific laboratory tests or biomarker that can distinguish patients who will relapse from those who would have a prolonged clinical remission after discontinuing AIT [56]. Therefore, the duration of AIT should be decided by the pediatric allergist on an individual basis taking in account benefits, possible risks and the agreement of the patient/family. The clinical improvement is closely related to the adherence to AIT. Allergen-specific immunotherapy is a prolonged treatment, thus educating the allergic child and his/her family remains a basic aspect to increase the adherence [48, 57, 58].

### Recommendation 3

The treatment should last at least 3 years (IbA). More prolonged treatment gave little adjunctive benefit in adults (Ib). Adherence is a pre-requisite for the AIT efficacy. Pediatric allergist must inform and properly educate children and their families in order to obtain the best possible adherence (IbA).

## Safety aspects

The absolute contraindications to AIT are concomitant malignancies, severe immune-associated diseases (e.g. severe immunodeficiencies), chronic and invalidating disorders and uncontrolled (symptomatic) asthma [44]. The safety of SCIT in children was evaluated in several systematic reviews. According to the available literature, systemic side effects are rare, but more frequent with SCIT than with SLIT [59–61]. The more recent systematic reviews [61] reported a high rate of local side effects for SLIT, but almost always mild, self-resolving and short-lasting. The local reactions usually were oral pruritus and swelling, oedema of lips, local paresthesia, abdominal pain, and diarrhea. Systemic reactions (urticaria, asthma, conjunctivitis) were anecdotal among SLIT patients and no fatality was described so far.

The lack of a standardized and homogeneous grading system in reporting and classifying the adverse events among the different studies is actually a strong limitation to define the real severity of each reaction. Nevertheless, a grading system to classify the adverse events of both routes of administration has been suggested [60, 61] (Tables 4 and 5), also according to the MedDRA definitions [62].

**Table 4:** Grading of systemic side effects (SCIT and SLIT) (simplified from 59)

GRADE 1	GRADE 2	GRADE 3	GRADE 4	GRADE 5
Symptoms/signs of one organ/system ● Cutaneous: generalized pruritus, urticaria, flushing. OR ● Angioedema (not tongue, laryngeal or uvular) OR ● Upper respiratory: rhinitis, cough OR ● conjunctivitis	Symptoms/signs of one organ/system OR ● Lower respiratory: asthma, cough with wheezing, chest tightness, shortness of breath. Fall <40% PEF, responding to SABA OR ● Gastrointestinal: abdominal cramps, vomiting, or diarrhea OR ● Uterine cramps	● Lower respiratory Asthma (eg, >40% fall PEF or FEV1) NOT responding to an inhaled bronchodilator OR ● Upper respiratory: laryngeal, uvula, or tongue edema with or without stridor	● Lower or upper respiratory: respiratory failure with or without loss of consciousness OR ● Cardiovascular: hypotension with or without loss of consciousness	Death

Patients may also have a feeling of impending doom, especially in grades 2, 3, or 4. Note: Children with anaphylaxis seldom convey a sense of impending doom and their behavior changes may be a sign of anaphylaxis; eg, becoming very quiet or irritable and cranky. Scoring includes a suffix that denotes if and when epinephrine is or is not administered in relationship to onset of symptom(s)/sign(s) of the SR:a, 5 min; b, >5 min to 10 min; c: >10 to 20 min; d:>20 min; z, epinephrine not administered. The final grade of the reaction will not be determined until the event is over, regardless of the medication administered. The final report should include the first symptom(s)/sign(s) and the time of onset after the allergen immunotherapy administration and a suffix reflecting if and when epinephrine was or was not administered, eg, Grade 2a; rhinitis:10 min. Symptoms occurring within the first minutes after the administration may be a sign of severe anaphylaxis.

Final Report: Grade a–d, or z__________ First symptom(s)/sign(s)___________ Time of onset of first symptom_____________

**Table 5:** Local side effects from SLIT (simplified from 60)

DESCRIPTION	GRADE 1: mild	GRADE 2: moderate	GRADE 3: severe
Pruritus, swelling of mouth, tongue or lips; throat irritation; nausea; abdominal pain; vomiting; heartburn; uvular edema	Not troublesome AND No treatment required AND No discontinuation	Troublesome OR Requiring symptomatic treatment AND No discontinuation	Grade 2 AND treatment discontinued

Some general rules should be applied when using AIT:

- SCIT
  - Assess the patient’s general condition.
  - Do not administer AIT in case of exacerbation of asthma.
  - Do not administer AIT in case of severe exacerbations of rhinitis, urticaria, atopic dermatitis.
  - Consider dose adjustment or temporary discontinuation in the case of recent systemic reactions such as asthma, urticaria, and rhinitis.
  - Discontinue AIT on ascertained anaphylactic reaction.
  - Adjust temporary the dose in case of injection-site granuloma (mid-postero-lateral upper arm).
  - Based on common sense precautions, starting SCIT during the pollen season (when there is a risk of symptom exacerbation) should be avoided. Nonetheless, the recent literature reports that a co-seasonal initiation of AIT and the reduction of doses do not increase the risk of adverse events [63].
  - Consider a downward dosage adjustment during the peak pollen season if symptoms worsen.
  - SCIT must always be administered by a trained physician/nurse.
  - maintain close observation for at least half an hour after injection,
  Epinephrine remains the treatment of choice in the case of moderate-severe immunotherapy-induced systemic reactions. This treatment should be always available, since the delayed administration of adrenaline is one risk factor for fatalities during anaphylaxis [64]. In children, intramuscular injection in the lateral thigh (vastus lateralis muscle) of adrenaline (aqueous epinephrine 1:1000 dilution [1 mg/ml]: 0.01 ml/Kg; maximum 0.5 mg/dose (=0.5 ml) with a 5-min interval between injections, as necessary) provides rapid bioavailability with a peak plasma concentration within 10 min, higher safety profile and longer duration of action.
  Further equipment rapidly available should be oral, intravenous/intramuscular H1 antihistamines and corticosteroids; inhaled salbutamol with spacer, oxygen 6–8 L/min by ventimask, intravenous fluid support with rapid infusion (10–15 min) of normal saline up to 5–10 ml/Kg. Intramuscular adrenaline must be always available.
- SLIT
  - Assess the patient’s general condition.
  - Do not administer AIT in case of exacerbation of asthma.
  - Do not administer AIT in case of severe exacerbations of rhinitis, urticaria, atopic dermatitis.
  - Consider temporary discontinuation of the treatment in case of any mouth ulcers or dental procedures.
  - Carefully evaluate if commencing an immunotherapy treatment during the pollen season in patients with pollen allergy.
  - Provide patients and their parents clear information about how to self-administer SLIT.

### Recommendation 4

Allergen specific immunotherapy in children is well tolerated (Evidence level: Ia). In clinical practice the management is different in case of perennial (mites) or pollen allergens. Immunotherapy can be initiated also in pre-school children if indicated. Indications are similar to those established for other age groups (III B).

## Long term and preventive effects of allergen specific immunotherapy

The natural history of respiratory allergies (allergic rhinitis and asthma) usually follows a reproducible pathway, the so called “atopic march” [65]. Concerning respiratory allergy, rhinitis is a well-known independent risk factor for the development of asthma and the bronchial hyperreactivity which is often an accompanying symptom represents an additive risk factor [66–70].

Several experimental data suggest that AIT can modify the natural history of allergic respiratory diseases. The effectiveness in altering the natural course of respiratory allergies was historically documented in an observational study [71]. After 14 years, children having received SCIT developed less asthma than children treated with pharmacotherapy alone. After more than 40 years, the Preventive Allergy Treatment (PAT) study [72], a prospective randomized controlled trial, tried d to evaluate whether SCIT, compared to pharmacotherapy alone, could prevent the development of asthma in children with seasonal allergic rhino-conjunctivitis due to grass pollen and/or birch allergy. The study showed that children treated with SCIT had a reduced risk of developing asthma with a significant positive odds ratio (OR). Indeed after 3 years of study, among the 151 children without asthma at the enrollment, 19/79 of the SCIT patients developed asthma versus 32/72 of the control group. This preventive effect was still present 2 and 7 years after discontinuation of the treatment, supporting the hypothesis of a preventive effect of AIT [73, 74]. However, some important methodological criticisms need to be disclosed, such as the lack of the double-blind design, the presence of children with asthma during the enrollment phase, the absence of data on the severity of asthma of the control group. Two other randomized trials investigated the preventive effect of AIT on the development of asthma. The first study evaluated 99 children aged 5 and 14 years with allergic rhinitis due to grass pollen [75]. After 3 years 18 of the 44 children in pharmacotherapy (controls) had developed asthma, compared to eight of the 45 children treated with SLIT. In this study, the risk of developing asthma resulted 3.8 fold increased in controls respect to SLIT treated children. Another study [76] evaluated 196 of 216 enrolled children aged 5 and 17 years with allergic rhinitis with or without intermittent asthma. Participants were randomized into two groups with a proportion of 2:1 for SLIT versus pharmacotherapy alone. After 3 years, the onset of persistent asthma was recorded in 2/130 children (1.5%) of the SLIT group compared to 19/66 (30%) of the control group (OR 0.04; 95% CI, 0.01–0.17). AIT was demonstrated to have a preventive effect also on bronchial hyper-reactivity. A randomized double-blind placebo controlled trial enrolling 30 children with asthma due to Parietaria showed that SLIT has a preventive effect on bronchial hyper-responsiveness to methacholine test during the pollen season: in the active group the degree of bronchial reactivity during the second pollen season was similar to the baseline values [77].

Currently, two double-blind placebo-controlled randomized studies were set up focusing the preventive effect of AIT on the development of asthma in children. The first one, on primary prevention of respiratory allergy was interrupted due to the poor adherence of younger children and problems related to ethical committees [78]. In the second study, children aged between 5 and 14 years with allergic rhinitis (but not asthma) due to grass pollen were randomized to receive treatment with SLIT or placebo. The aim of this study, currently ongoing is to evaluate the development of asthma after 5 years from the beginning of AIT [79]. The results of these preventive intervention studies will provide evidences on the effective role of AIT in the primary and secondary prevention of allergic diseases. However, asthma is well recognized as a multifactorial disease with characteristics that depend on many variables, such as infections, bronchial reactivity, allergy, indoor and environmental pollution, comorbidities, which have led in the recent years to a better identification of different phenotypes and endotypes of this disease. Therefore, further RCTs are needed to verify the preventive effect of AIT on asthma onset in children with allergic rhinitis and/or IgE-associated atopic dermatitis.

As for the risk of developing new allergic sensitizations over the years, it is well known that the sensitization may occur in the first years of life, frequently with IgE production against foods, then against environmental allergens (house dust mites) and finally pollens, such as grasses, Parietaria or birch. Nevertheless, many children may first experience allergy to mites, without presenting a previous food allergy; and, also the sensitizations to pollens or molds like Alternaria may appear without prior allergies [80, 81]. There is evidence suggesting that the preventive effect of AIT on the natural history of allergic respiratory diseases does not affect only the progression towards asthma, but also on the appearance of new sensitizations. In one study, 22 children aged 4–6 years and monosensitized to house dust mites were treated with SCIT and pharmacotherapy and compared with 22 peers treated with pharmacotherapy alone. After 3 years, 10/22 children (45%) of the SCIT group did not show new sensitizations, while all of the 22 controls developed one or more new sensitivities [82]. Another non-randomized prospective study provided similar results [83]. Seventy-five of 138 children (aged 5–8 years) monosensitized to mites affected by intermittent asthma were treated for 3 years with SCIT and pharmacotherapy and the remaining with pharmacotherapy alone controls. Three years after the discontinuation of SCIT, 75.4% of children in the SCIT group did not develop new sensitizations, while only 33,3% of the controls remained monosensitized.

Several studies conducted in the last 20 years have documented that the clinical benefits of AIT can last long after the discontinuation of the treatment [7]. The long-term effects have been observed for both SCIT and SLIT, and represent an exclusive prerogative of AIT. Indeed, none of the currently available pharmacological treatments for respiratory allergies has shown a comparable long lasting effectiveness after its discontinuation, nor a potential in modifying the natural history of these diseases [55, 84–88]. The results of controlled clinical trials carried out with both routes of administration (subcutaneous and sublingual) have confirmed that AIT, as etiological therapy for IgE-associated respiratory diseases, can maintain improvement in symptoms even after its discontinuation. Also in these cases the studies are heterogeneous in duration, patient selection, and largely focused on subjects allergic to grasses. To date, evidence on preventive and long-term effects of AIT is suggestive, but not definitive yet.

### Recommendation 5

Despite of the methodological limitations, the currently available studies have shown that an early therapeutic intervention with AIT, when the child is at the early stages of the respiratory disease and still mono- or pauci-sensitized, can be evaluated as therapeutic option in the clinical practice. The long-term efficacy after the discontinuation of the treatment can be considered as a unique and distinctive characteristic of AIT (IIaB).

## Allergen-specific immunotherapy for food allergy (FA)

FA remains a growing health issue, with an estimated prevalence of about 6–8% in the pediatric population [89–92]. Nonetheless, estimating the true prevalence of FA remains difficult, depending on the local nutritional habits, different phenotypes, and unavoidable inaccuracy in diagnosis. The definition of FA assumes that symptoms caused by the ingestion of a food represent the effect of an immune response, which can result either from IgE- or non-IgE mediated mechanisms or a combination of both [91, 92]. The most frequently causative foods are cow’s milk (CM), hens’ egg (HE) and peanut, distantly followed by fish, shellfish, meat and wheat. FA should be suspected on medical history and results of in vivo and in vitro allergy tests, but oral food challenge, possibly in a double blind fashion, still remains the cornerstone in the diagnostic process. The only effective treatment is the complete allergen avoidance in association with the use of emergency medications to be used when accidental reactions occur. However, a complete elimination diet is difficult to perform, especially for common foods as CM and HE, which can be present in food as hidden allergens. All these aspects heavily affect the quality of life of children and caregivers [93–96]. In this context, considering the potential desensitizing effects of allergen administration, several therapeutic strategies targeting the IgE-associated FA reactions were investigated, including oral immunotherapy (OIT) and SLIT, to achieve desensitization. OIT and SLIT for FA involve the administration of slowly increasing doses of the allergen source until the food is tolerated at usually dietary doses. This approach can confer protection against accidental allergic reactions and contribute to improve nutritional status and quality of life of the affected patients [97]. It is not clearly defined if when desensitization has been achieved, a permanent tolerance persists, independent of the regular ingestion of the responsible food.

Many clinical trials performed with cow milk, egg and peanuts are nowadays available, as reviewed in numerous articles [98–107]. All the published trials (Tables 6, 7 and 8), consistently show that an effective desensitization can be obtained in 60–80% of children, that about 10% of children undergoing desensitization must stop the procedure due to severe adverse events and that a permanent (or long-lasting) tolerance is achieved in about 50% of the patients with a previous successful desensitization [108]. Oral or sublingual desensitization procedures for food allergy still remain an experimental model to be used only under medical supervision and/or in research context [109, 110].

**Table 6:** Summary of the RCTs with cow’s milk

Study (Author, year, country)	Design	Active group vs comparator	Sample size (AG/CG)	Age (yrs) [mean (range)]	Duration desensitization protocol/maintenance dose	Outcomes: desensitization/sustained unresponsiveness	Discontinued due to AEs	SAEs
Caminiti, 2009, Italy [125]	RCT [DBPCRT (6 pts); open fashion (7 pts)]	OIT vs placebo	10/3	8 (5–10)	18 weeks/200 ml	AG: 7 pts complete OD (200 ml of CM); 1 pt partial OD (64 ml of CM)./CG: none spontaneously tolerant, [OFC (+)]	2	In AG, 3 SAEs: two withdrawals (both severe anaphylaxis:one including shock, the other laryngeal edema); 1 pt with partial tolerance (generalized urticaria-angioedema, cough)
Longo, 2008, Italy [126]	RCT	OIT vs routine care (food avoidance)	30/30	8 (5–17)	1 year [rush build-up phase (10 days) + maintenance phase]/150 ml	AG: 11 pts (36%) complete OD (≥150 ml CM); 16 (54%) partial OD (5–150 ml)/CG: none spontaneously tolerant [OFC (+)]	3	Rush phase: i.m. E four times in 4 pts; nebulized E in 18 pts and more than once in 7 pts. Slow (home) dosing: 2 pts treated in the emergency department (CS, AH), and i.m. E (1 case).
Martorell, 2011, Spain [127]	parallel-group, multicentre RCT	OIT vs routine care (food avoidance)	30/30	2.2 (2–3)	1 year [build-up phase (16 weeks) + maintenance phase]/200 ml	AG: 90% complete OD. Two withdrawals; one partial OD (35 ml of CM)/CG: 23% pts natural tolerance [OFC (+) in 3/23 pts]	1	None
Pajno, 2010, Italy [128]	Random single-blind controlled study	OIT vs placebo	15/15	9 (4–10)	18 weeks/200 ml	AG: 10 pts complete OD (200 ml of CM) and in 1 pt partial tolerance (100 ml)/CG: none spontaneously tolerant, [OFC (+)]	2	2 SAEs requiring i.m. E in 2 pts (withdrawals)
Skripak, 2008, USA [129]	DBPCRT	OIT vs placebo	13/7	9.3 (6–21)	23 weeks/500 mg	Median OFC threshold increased from 40 to 5140 mg after OIT in AG/all pts in the PG reacted at 40 mg	1	Median frequency of SAEs (E use): 1% (0.2%) of active doses vs none in the placebo group

*AG* active group, *AH* antihistamines, *CG* control group, *CM* cow’s milk, *CS* corticosteroids, *E* epinephrine, *Pt* participants, *SAE* severe adverse event

**Table 7:** Summary of RCTs with hen egg

Study (Author, year, country)	Design	Active group vs comparator	Sample size (AG/CG)	Age (yrs) [mean (range)]	Duration desensitization protocol/maintenance dose	Outcomes: desensitization/sustained unresponsiveness	Discontinued due to AEs	SAEs
Burks, 2012, USA [130]	RCT	OIT vs placebo	40/15	7 (5–11)	22 months/1.6 g DEW	After 22 months, 30 (75%) pts in AG passed 10-g- OFC/at 6–8 weeks later only 11 (28%)	5	No reports of severe or life-threatening symptoms or death
Caminiti, 2015, Italy [131]	DBPCRT	OIT vs placebo	17/14	6 (4–11)	4 months/4 g DEW	AG: 16/17 pts (1 dropout) complete OD/AG: After 3-months-HE-avoidance, 31% of these 16 pts remained tolerant. CG: only 1 pt passed the final OFC.	1	3 SAEs: during OD, 1 pt [withdrawal (U, throat pruritus, R, A, vomiting); during HE-containing diet: 1 pt presented U, abdominal pain after exercise (1 cooked HE) and another wheezing and cough during upper respiratory infection (1 cooked HE). [Both tolerant after 3 months of HE containing diet discontinuation]

*A* asthma, *AE* adverse event, *AG* active group, *CG* control group, *DBPRT* Double blind placebo controlled randomized trial, *DEW, E* epinephrine, *HE* Hens’ egg, *OD* Oral desensitization, *OFC* oral food challenge, *OFS* oropharyngeal symptoms, *OIT* Oral immunotherapy, *OT* Oral tolerance, *Pt* participant, *R* rhinitis, *RCT* randomized controlled trial, *SAE* severe adverse event, *U* urticaria, *wk* week

**Table 8:** Summary of RCTs with peanut allergen

Study (Author, year, country)	Design	Active group vs comparator	Sample size (AG/CG)	Age (yrs) [mean (range)]	Duration desensitization protocol/maintenance dose	Outcomes: desensitization/sustained unresponsiveness	Discontinued due to AEs	SAEs
Fleischer, 2012, USA [132]	DBPCRT, multicentre crossover trial	SLIT vs placebo	20/20	15 (12–37)	Phase 1, DBPCRT (SLIT vs placebo): 44 weeks/maintenance dose: 1386 μg/day of peanut protein. Phase 2, After an unblinding 5-g DBPCFC, pts in AG continued on maintenance dosing with a 10-g OFC after 1 year of maintenance therapy and placebo pts crossed over to active peanut SLIT (max maintenance dose of 3694 μg). A 5 g crossover OFC was performed after 44 weeks of SLIT.	Week 44 (Unblinding 5 g-OFC): OD: 70% (*n* = 14) in AG vs 15% CG (*p* < 0.001). Median OFC threshold increased from baseline significantly (371 vs 21 mg) only in AG (*p* < .01). Week 68 All Week 44 responders were still responders with further significant increase in median OFC threshold (996 mg) than at Wk 44 (*p* = .05) and baseline (*p* = .009)	1	Only one out of 127 AEs required E and oral antihistamine
Kim, 2011, USA [133]	DBPCRT	SLIT vs placebo	11-lug	5.2 (1–11)	1 year [build-up phase + maintenance phase (6 months)]/2000 μg of peanut protein	Median cumulative dose safely reached at OFC increased 20-fold (up to 1710 mg, 6–7 peanuts) in AG/In CG, 85 mg (<1 peanut) [OD: AG vs CG, *p* = 0.011]	None	No E required for whole study.
Tang, 2015, Australia [134]	DBPCRT	(OIT + probiotic, Lactobacillus VS placebo	31/31	6 (1–10)	18 months [build-up phase (8 months) + maintenance phase (10 month) + peanut elimination diet (median 2.3 weeks, range, 2–5.3 weeks)/2 g peanut protein	After OIT, AG: 87% OD/After OIT+ peanut elimination diet, AG: possible sustained unresponsiveness in 82.1%; CG: 3.6% spontaneous tolerance (*P* < .001)	none	AG: 45.2% CG: 32.3% Number of SAEs per pt did not differ by group (*P* = .9).
Varshney, 2011, USA [135]	DBPCRCT	OIT vs placebo	19-set	6 (2–10)	148 weeks/4 g	5 g-OFC, AG: 84% complete OD/CG: 100% pts ingested a median cumulative dose of 280 mg at OFC (range, 0–1900 mg) [*p* < 0.001].	3	At DBPCFC 0/16 in AG required E, 3/9 in CG

*AG* active group, *CG* control group, *DBPRT* Double blind placebo controlled randomized trial, *LRs* Local reactions, *OD* Oral desensitization, *OFC* oral food challenge, *OIT* Oral immunotherapy, *OT* Oral tolerance, *Pt* participant, *RCT* randomized controlled trial, *SAE* severe adverse event

### Recommendation 6

Based on current evidence, we recommend to perform food OIT/SLIT only in highly specialized centers and under strict medical supervision after the informed consent has been obtained from parents (IaA).

## Allergen-specific immunotherapy for “extrinsic” atopic dermatitis (AD)

AD is a chronic inflammatory skin disease deriving from a complex interaction among genetic factors, impaired skin barrier function, immune dysregulation and exposure to various environmental allergens and infectious agents [111, 112]. Most patients have increased total IgE and are sensitized to aeroallergens (mainly house dust mite), this defining the “extrinsic” form of AD. In such cases, the exposure to allergens may exacerbate the disease [110, 111]. It is still controversial whether AIT may be beneficial for patients with AD. Considering the clinical effectiveness of AIT in IgE-associated allergic diseases, several trials, often uncontrolled, investigated the efficacy of AIT for patients with “extrinsic” AD and reported overall controversial results [9, 113, 114]. It is true that some proofs of clinical efficacy in extrinsic AD are available [114, 115], but no clear indication or recommendation can be made. Also in this case, the use of AIT in AD remains experimental, and the indications are confined to allergic rhinitis/asthma, when concomitant AD is present.

### Recommendation 7

The efficacy of AIT in children and adults with AD is still controversial. A more precise selection of clinical phenotypes (e.g., presence of IgE-sensitization to house dust mites, concomitant respiratory allergy, evidence of a cause-effect relationship between IgE-sensitization and AD exacerbation) may help identifying patients with extrinsic AD who could benefit from AIT (Evidence Level: III).

## Regulatory aspects of allergen-specific immunotherapy

The European Academy of Allergy and Clinical Immunology (EAACI) has recently published a declaration which supports the key role of AIT in the treatment of IgE-associated allergic diseases and calls upon Europe’s policy-makers to promote awareness of the effectiveness of AIT and funding for AIT research. For many years most allergen products have been marked in European countries as “named patients products” (NPP), which only require to be prepared in compliance with Good Manufacturing Practice to get a marketing authorization. In addition, NPP don’t require independent evaluation of quality, efficacy, and safety and there is no demand for manufacturer to notify adverse events. The regulatory scenario for AIT changed with the Directive 2001/83/EC and 2003/63/EC of the European Parliament which recognize that: 1) allergens are medicinal products capable to identify or induce a specific acquired alteration in the immunological response to a sensitizing agent; 2) as medicines produced with an industrial process, allergens require a marketing authorization (AIC) in Europe according to the procedures established for all drugs (i.e., centralized, mutual recognition, decentralized, or national) and following a clinical development through all phases of RCTs [14, 116]. The European Medicines Agency (EMA) has recently released specific guidelines for designing clinical studies on the development of products for AIT [117]. Clinical trials in the pediatric population should also follow the EMA pediatric investigation plan (PPI) ([118], https://www.clinicaltrialsregister.eu/ctr-search/trial/2012-005678-76/IT). However the Directive 2001/83/EC of the European Parliament has also established some exceptions (e.g. prescription for individual patients under the direct doctor responsibility) which still allowed the use of allergen products as NPP instead of registered medicines (https://www.clinicaltrialsregister.eu/ctr-search/trial/2012-005678-76/IT). Furthermore, in Europe every pharmaceutical manufacturer provides its standardization of allergen extracts for AIT. This legitimate assertion of autonomy in research and production of AIT have resulted in significant discrepancy in the allergen concentration in extracts of different manufacturers.

The use of registered products for AIT in the pediatric population could represent a new era for patients suffering from IgE-associated allergic diseases. The allergen manufacturer should provide adequate scientific documentation of efficacy and safety of their products with the aim to improve quality and standardization of allergenic extracts. Therefore AIT extract should be set up with high quality product, properly standardized and containing sufficient doses of allergens [119].

For all these reasons the Italian Medicines Agency (AIFA) in October 2009 organized a Working Group on allergens, with the aim to: 1) verify the existence of an acceptable level of quality, efficacy and safety for allergen products currently on the market as NPP; 2) define the registration process for allergenic extracts. To facilitate this regulatory process, the AIFA is currently funding an independent multicenter, prospective DBPC randomized trial to evaluate the efficacy, safety, tolerability and cost-effectiveness of SLIT to house dust mite in combination with standard of care in pediatric allergic asthma.

## The unmet needs and the future directions

Data from systematic reviews of meta-analyses ascertained the efficacy of AIT in the treatment of allergic respiratory diseases [27]. Despite this, some methodological criticisms were evidenced: a) the amount of the administered maintenance dose is largely variable and established only for few preparations [27, 60]; b) the protocols of administration are neither univocal nor standardized; c) the description and classification of side effects is variable among studies, and between controlled trials and post marketing surveys; d) more detailed studies with objective parameters measured are needed in asthma [120]. All these aspects make difficult to compare each study with another. In addition, the duration of treatment (that varies from 6 to 36 months) and the regimen of administration are not well standardized. Finally, there is still a discrepancy in the standardization methods, and the content of major allergen(s) remains largely variable among manufacturers [121, 122].

There is still room for improving AIT as far as prescription, efficacy and safety are concerned. The molecular-based diagnosis would certainly improve the accuracy in AIT prescription [22, 123, 124], allowing to dissect the genuine sensitizations and the cross-reactions due to pan-allergens. The epicutaneous and intralymphatic routes of administration would represent an intriguing prospective approach, especially in the pediatric age [27]. Nonetheless, all these approaches remain experimental.

## Allergen specific-immunotherapy in pediatric clinical practice (Fig. 1)

A crucial issue with AIT in clinical practice is continuous communication with children and their families in order to maintain compliance to such a long-term treatment program, which lasts 3 to 5 years. Adolescents, in particular, should be properly motivated.

It is necessary to specify that SCIT requires considerable efforts for the first months of treatment. Later on, in most cases, the subcutaneous administration has a monthly schedule and it is more easily accepted. Currently on the market there are “allergoids”, preparations which greatly reduce the commitment of subcutaneous administration.

As for SCIT, patients and families should be completely aware about the mode of administration of SLIT. After the first administration, which must be made in a controlled setting, SLIT is continued at home, therefore parents should check the regular intake of SLIT by their child.

Regardless of the route of AIT administration, parents must be informed on the cost, possible side effects and expectations of AIT effectiveness. Once started, AIT must be conducted regularly, arranging for periodic visits and frequent alerts to the family in order to maintain compliance. Based on the clinical data available, it can be assessed with the family whether to start a course of AIT even for milder cases of IgE-associated allergic diseases with the primary aim to prevent the development of asthma or new sensitizations. Currently there are no indications to prefer SLIT over SCIT. The advantages of SLIT are a greater safety profile and an easier administration route which does not require the presence of a doctor or a qualified health professional except for the first dose. The allergen choice must be based on careful medical history, results of allergy diagnostic tests and demonstration of a correlation between the identified allergen and symptoms. In case of poly-sensitized patients, the causal role of each individual allergen needs to be established in order to choose those most clinically relevant.

For AIT treatments, the instructions given by the manufacturer of the commercial extracts should be followed. As a general rule, AIT involves an induction phase (build-up) followed by a maintenance phase. SCIT must be performed by a doctor or another healthcare professional in a hospital or in a controlled setting, fully equipped to deal with emergencies. Readily available are required: adrenaline in vials or self-injectors, oxygen by mask or nasal cannula, corticosteroids for intravenous administration and a bag valve mask for ventilation. If an adverse event occurs, the child must be stabilized first and transferred to a pediatric emergency room only when table. The SCIT induction phase should start with low doses. There are different protocols for SCIT induction phase which comprise different timing ranging from days or weeks (rush and ultra-rush schemes) to months. Once the maintenance dose is reached, it is commonly administered every 2 weeks for at least 3 months, and afterwards monthly After every subcutaneous injection, patients should remain under observation for at least 30 min; furthermore, physical exercise should be avoided in the following hours. The side of SCIT injections is usually the side anterior or rear surface of the arm.

Concerning SLIT, the first dose should be administered in a hospital or clinic under medical supervision. The SLIT induction phase is usually shorter than that of SCIT, ranging between 3 and 5 days. It is important to specify that drops or tablets must be kept under the tongue for 2 min and then swallowed. It is advisable that patients keep a symptom diary to record all reactions apparently related to the assumption of SLIT. A fix and simple schedule of administration would increase the compliance to the treatment.

Dosage reduction or temporary interruption of AIT may be justified in the case of:

- acute asthma exacerbation;
- concurrent febrile illness;
- moderate to severe adverse events;
- prolonged treatment discontinuation.

Treatment can be resumed with the same maintenance dose, if the suspension was relatively short (up to 2 weeks). In the event of more prolonged interruption, AIT should be resumed by reducing the dose or restarting the induction phase with the same initial conditions if any. Uncontrolled asthma remains the major absolute contraindication to both SCIT and SLIT [44].

Clinical parameters like exacerbation of symptoms or use of relief medications can be helpful in evaluating the effectiveness of AIT. In addition, pulmonary function tests are crucial in evaluating children with asthma or assessing the involvement of the lower airways in children with allergic rhinitis. The repetition of skin test or specific-IgE assay cannot replace, in clinical practice, the physician’s judgment about the patient status undergoing AIT.

If a clinical improvement along with significant reduction in the use of medications is not observed after 1 year of therapy, the indication for AIT must be revaluated. It is therefore necessary to reassess the appropriateness of the selection of the allergic child and eventually the diagnosis of respiratory allergy. After the end of treatment, children should be checked at least once every year to verify the long-term effectiveness of AIT.

## Conclusions

AIT is the unique aetiological and immune-modifying treatment currently available for patients suffering from IgE-associated diseases. It should be considered in pediatric patients, also preschool children, with rhino-conjunctivitis and/or asthma with an ascertained IgE-associated respiratory allergy, caused by clinically relevant allergens. The decision to start the treatment depends on several factors. It should be always used in association with appropriate pharmacotherapy and for at least 3 years. AIT is not only well tolerated but also efficacious in the short and long-term and in prevention of new sensitizations and development-impairment of asthma. Concerning food allergies, based on current evidence, we recommend to perform food OIT/SLIT only in highly specialized centers and under strict medical supervision after the informed consent has been obtained from parents. OIT/SLIT standardized products are awaited. The efficacy of AIT in children and adults with extrinsic AD is still controversial and needs further large and well-designed studies.

## Abbreviations

AD: Atopic dermatitis
AIT: Allergen ImmunoTherapy
CM: Cow milk
DBPC: Double-blind, placebo controlled
EA: Egg allergy
EPIT: Epi-cutaneous immunotherapy
FA: Food allergy
NPP: Named patients product
OIT: Oral immunotherapy
PA: Peanut allergy
SCIT: Subcutaneous immunotherapy
SLIT: Sublingual immunotherapy
